# Gene selection using pyramid gravitational search algorithm

**DOI:** 10.1371/journal.pone.0265351

**Published:** 2022-03-15

**Authors:** Amirhossein Tahmouresi, Esmat Rashedi, Mohammad Mehdi Yaghoobi, Masoud Rezaei

**Affiliations:** 1 Faculty of Medicine, Kerman University of Medical Sciences, Kerman, Iran; 2 Department of Electrical and Computer Engineering, Graduate University of Advanced Technology, Kerman, Iran; 3 Department of Biotechnology, Institute of Science and High Technology and Environmental Sciences, Graduate University of Advanced Technology, Kerman, Iran; Torrens University Australia, AUSTRALIA

## Abstract

Genetics play a prominent role in the development and progression of malignant neoplasms. Identification of the relevant genes is a high-dimensional data processing problem. Pyramid gravitational search algorithm (PGSA), a hybrid method in which the number of genes is cyclically reduced is proposed to conquer the curse of dimensionality. PGSA consists of two elements, a filter and a wrapper method (inspired by the gravitational search algorithm) which iterates through cycles. The genes selected in each cycle are passed on to the subsequent cycles to further reduce the dimension. PGSA tries to maximize the classification accuracy using the most informative genes while reducing the number of genes. Results are reported on a multi-class microarray gene expression dataset for breast cancer. Several feature selection algorithms have been implemented to have a fair comparison. The PGSA ranked first in terms of accuracy (84.5%) with 73 genes. To check if the selected genes are meaningful in terms of patient’s survival and response to therapy, protein-protein interaction network analysis has been applied on the genes. An interesting pattern was emerged when examining the genetic network. HSP90AA1, PTK2 and SRC genes were amongst the top-rated bottleneck genes, and DNA damage, cell adhesion and migration pathways are highly enriched in the network.

## 1. Introduction

Classification of high-dimensional microarray gene expression data is a major problem in bioinformatics. From biological perspectives, a large proportion of the genes are redundant for classification. By gene selection (GS), the accuracy could be improved. Soft computing and machine learning techniques could be promising for finding the most informative and predictive genes.

Engineers and mathematicians widely investigate gene selection for disease classification (primarily malignancies). Lung, breast, and prostate cancers are some of the extensively investigated malignancies. Cancer is an abnormal growth of cells caused by multiple genetic aberrations leading to a dysregulated cell proliferation. Tumors often have distinctive gene expression profiles which could be useful for diagnosis and prediction of response to therapy.

Breast cancer is the most frequent cancer diagnosed in women and the second leading cause of cancer mortality in developed countries [1]. The methods now commonly employed to categorize patients are mainly based on immunohistochemistry (IHC) staining. However, in certain situations, these methods are not adequately precise to estimate the prognosis of patients or response to therapy [2]. Over the past decade, a considerable effort has been dedicated to categorize patients with breast cancer into subtypes that might influence therapeutic decisions [3]. PAM50 is a gene expression-based predictor panel that is developed to classify patients into four subgroups by quantitative measurement of fifty genes robustly correlated with IHC staining [4]. Nevertheless, the efficacy of PAM50 in predicting the prognosis of triple-negative breast cancer (TNBC) individuals still remains a matter of debate [5].

There has been a growing interest in employing machine learning methods for high-dimensional feature selection (FS) problems. In a bird’s eye view, there are three principal approaches for FS in classification tasks. Filter-based, wrapper, and hybrid methods. Filter-based also known as statistical methods, only consider one or a combination of statistical aspects of data for feature selection. For instance, features with high entropy or low redundancy values, and high discriminative power. Wrapper methods work jointly with a classifier and try to find the features with maximum classification accuracy. Hybrid approaches take the advantage of both filter and wrapper methods. Feature selection problems are NP-hard; So, heuristic random search algorithms are a suitable proposition. They could find the sub-optimal solutions in complicated and large problems, and in some cases, they are more accurate and applicable than filter-based methods. Inspired by a random search algorithm, these methods try to select the best subset of features. In feature selection using heuristic search algorithms, the goal is maximizing classification accuracy [6].

Gravitational search algorithm (GSA) is a meta-heuristic optimization algorithm inspired by law of gravity and mass interaction [7, 8]. GSA and its derivatives, were employed in solving various engineering problems like function optimization [7–10], feature selection [11–15], image processing [12, 14, 16], and circuit design [17, 18].

In this paper, a pyramid version of GSA is used for solving high-dimensional gene selection problems. The proposed method is a hybrid approach that cyclically reduces the number of genes and selects the least genes for achieving high classification accuracy. The term pyramid as depicted in the graphical abstract, indicates the down-sloping process of feature selection using PGSA in which the depth of the pyramid is determined by the nature of the problem and number of features whom might needed.

This paper is organized as follows. Reviewing the related works is presented in Section 2. The proposed method for gene selection is introduced in Section 3. The comparison results are discussed in Section 4. Finally, the paper is concluded in Section 5.

## 2. Previous works

Filter-based methods rank the features based on the statistical properties and select high-rank features. These properties are mutual information, entropy, information gain, F1-score, Chi-square, and correlation. Filter methods do not use learning algorithms [19]. Filter and wrapper methods for gene selection are reviewed in [20]. Some researchers used heuristic search algorithms [6, 21]. A recently published systematic review [22] has performed a thorough study on feature selection algorithms on gene expression microarray data and they found that hybrid FS methods were the most captivating method in microarray FS problems. The majority of statistical methods are faster and simpler than machine learning-based methods. Nevertheless, the major drawback of them is ignoring the interactions between features in classification.

In [21], a novel ant colony optimization algorithm, incorporated with a filter method was produced for gene selection to minimize gene redundancies. The hybridization of the genetic algorithm (GA) and artificial bee colony (ABC) was produced in [6] for gene selection; The support vector machine (SVM) was employed for classification. A classifier by hybridization of cuckoo optimization algorithm (COA) and genetic algorithm (GA) was introduced in [23], which selected the meaningful genes in cancer classification using shuffling; SVM and multi-layer perceptron (MLP) was used as the classifier.

A variant of moth-flame optimization (B-MFO) for binary classification problem is developed by [24] using three different transfer functions (sigmoid, hyperbolic and U-shaped) to convert the continuous MFO to fit for binary feature selection problem. Their findings show that transformation functions have a substantial impact on algorithm behavior when it comes to updating the position of search agents and finding the best solution to the feature selection problem.

Multi-trial vector-based differential evolution (MTDE) is a metaheuristic optimization algorithm that is based on a multi-trial vector search strategy (i.e., trial vector producers (TVPs)). In this algorithm, several subpopulations which are dispersed according to a winner-based policy are generated and TVPs are applied on their dedicated subpopulations then they communicate their experiences through a life-long experience [25].

Quantum-based avian navigation optimizer algorithm (for short, QANA) is inspired by the meticulous precision of birds during migration for long-distances [26]. In QANA, the population is distributed into multiple flocks to explore the search space utilizing a self-adaptive quantum orientation and two mutation mechanisms called DE/quantum/I and DE/quantum/II (in which DE means differential equations). The assignment of flocks is based on success-based population distribution (SPD). The information flow communicates through the population using V-echelon. In [27], a binary form of Sine Cosine Algorithm (SCA) has been generated for medical datasets using two V-shaped and S-shaped transform functions while the search space remained continuous.

A new variant of whale optimization algorithm (WOA) which consider the spatial boundaries has been proposed by [28] to solve the high-dimensional gene selection process. Modified cat swarm optimization (MCSO) was used in [29] to select the dataset’s most relevant features; SVM, kernel ridge regression, and random forest were used for classification. In [30], Grasshopper optimization algorithm (GOA) was used to simultaneously optimizing the SVM parameters and selecting best subset of features. In [31], a binary bat optimization algorithm adjunct with an extreme learning machine has been used to optimize a particular fitness function which computes a score for every feature and tries to maximize interclass distance and minimize within-class distance.

In [32], an improvised interval value-based particle swarm optimization (PSO) algorithm implemented to select the best genes for cancer classification. In [33], the gene numbers were reduced by Fisher criteria followed by a wrapper gene selection algorithm using cellular learning automata and ant colony search algorithm for gene selection to increase the classification accuracy.

In [34], binary particle swarm optimization (BPSO) and gene-to-class sensitivity information were used to select genes and improve accuracy. An extreme learning machine was used to classify data [35] and to produce a hybrid gene selection algorithm by combining a filter FS method and Binary Differential Evolution (BDE) algorithm. In this method, firstly, features are ranked using the information gain. Then, high-ranked features are used for initializing the BDE population. BDE’s operators are performed, and the best set of features maximizes the classification accuracy with fewer features.

A forward gene selection algorithm was introduced in [36]. This method produces an augmented dataset to achieve good results in cases with few samples and a regression algorithm that selects the gene groups. The cost function in the regression algorithm is the sum of the squared errors with the L2-norm penalty function. Gene selection for autism using the aggregation of some feature selection methods was produced in [37]; In this method, SVM classifies the genes selected by different methods, and at the second stage, a random forest of decision trees is used to get the final decision.

A combination of support vector machine recursive feature elimination algorithm and support vector machine t-test recursive feature elimination was employed in [38]. T-score with sample selection was used in [39] for gene selection. T-score is based on t-statistics measuring the correlation between input features and output class labels. In this method, relevant samples are selected at each iteration using a modified logistic regression loss function, and then genes are ranked by computing T-score for these samples. A Maximum–Minimum Correntropy Criterion (MMCC) approach was introduced in [40] to select informative genes from microarray data. Correntropy locally measures the similarity between two random vectors, and it is defined as the expectation of the kernel function applied to these vectors. MMCC is a filter-based method, and after selecting genes, it uses SVM to classify data.

A modification of the analytic hierarchy process gene selection method by incorporating statistics of several gene-ranking methods, including two-sample t-test, entropy, ROC curve, Wilcoxon test, and signal to noise ratio, was presented [41]. Due to a smaller number of samples, leave-one-out was preferred to k-fold cross-validation. In [42], informative genes were selected using mutual information between genes and classes, and the disease was classified using selected genes and SVM. Integration of the partial least squares (PLS) based recursive feature elimination with simulated annealing and square root was produced in [43] and employed for gene selection.

A two-phase gene selection approach based on a combination of multivariate filter method and wrapper method, optimized by recursive binary GSA was utilized by [44]. A swarm intelligence-based search algorithm based on improved binary GSA and information gained has been applied on five cancer datasets [45]. They used the k-nearest neighbors’ algorithm with K = 1 and compared results with the locality-sensitive Laplacian score (LSLS) method. The proposed method outperformed the LSLS method in 4 of 5 datasets regarding accuracy, precision and recall. A hybrid wrapper method which is a combination of teaching learning-based algorithm (TLBO) and gravitational search algorithm (GSA), called TLBOGSA, was developed by [46]. In the first step of gene selection, minimum redundancy maximum relevance (mRMR) has been applied to the data and then a wrapper method tries to find the most informative genes. The GSA has been used in the teaching phase to improve search capability. The overall accuracy was above 98%.

## 3. Materials and methods

IBGSA [13] is an improved version of BGSA with *N* searcher objects (agents). The population of agents is initialized randomly. The *i*^*th*^ object is considered as a binary vector with the *D* dimensions as the following.

$$X_{i}=\left(x_{i}^{1},\ldots ,\;x_{i}^{d},\ldots ,x_{i}^{D}\right)$$ (1)

The goal is to find the object, which has produced the best objective value. Here, the classification accuracies are considered as the objective values. The mass of each object is defined as Eq 2.

$$M_{i}\left(t\right)=\frac{{\text{fit}}_{\text{i}}\left(\text{t}\right)-\text{worst}\left(\text{t}\right)}{\sum_{j=1}^{N}{\text{fit}}_{\text{j}}\left(\text{t}\right)-\text{worst}\left(\text{t}\right)}$$ (2)

$$M_{ii}=M_{ai}=M_{pi}=M_{i}$$

Where *M*_*i*_(*t*) and *fit*_*i*_(*t*) are the mass and fitness values of the *i*^*th*^ object, respectively, *worst*(*t*) is the population’s worst fitness. Total forces of the *K* heavier objects toward the other objects are computed using Eq 3, and the acceleration is reachable with Eq 4.

$$F_{i}^{d}\left(t\right)=\overset{}{\underset{j\in \text{Kbest},\;i\neq j}{\sum }}rand_{j}F_{ij}^{d}\left(t\right),\;F_{ij}^{d}\left(t\right)=G\left(t\right)\frac{M_{pi}\left(t\right)\times M_{aj}\left(t\right)}{R_{ij}{\left(t\right)}^{p}+E}\left(x_{j}^{d}\left(t\right)-x_{i}^{d}\left(t\right)\right)$$ (3)

$$a_{i}^{d}\left(t\right)=\frac{F_{i}^{d}\left(t\right)}{M_{ii}\left(t\right)}$$ (4)

The velocity of an object is updated by adding the obtained acceleration to a fraction of its current velocity as Eq 5.

$$v_{i}^{d}\left(t+1\right)=rand_{i}\times v_{i}^{d}\left(t\right)+a_{i}^{d}\left(t\right)$$ (5)

In binary environments, dimensions have values of 0 or 1. In IBGSA [13], the probability of switching from 0 to 1 or vice versa is carried out by a transfer function (*Tfn*), which is computed with the use of Eq 6. Then, a rule defined as Eq 7 is employed to obtain the positions of the objects.

$$Tfn\left(v_{i}^{d}\left(t\right)\right)=A+\left(1-A\right)\times \left|\text{tanh}\left(v_{i}^{d}\left(t\right)\right)\right|,\;A=k_{1}\left(1-exp\left(\frac{FC}{k_{2}}\right)\right)$$ (6)

$$\text{if}\;\text{r}\;\text{and}()<\text{T}\;\text{fn}(v_{i}^{d}(t+1))\;\text{then}$$ (7)

$$x_{i}^{d}\left(t+1\right)=complement\left(x_{i}^{d}\left(t\right)\right)$$

$$else\;x_{i}^{d}\left(t+1\right)=x_{i}^{d}\left(t\right)$$

Where k1 and k2 are constant parameters. Fc is the failure counter. A failure happens if the best-found solution does not change after one iteration. If failure occurs, Fc increases by one and if success occurs, Fc is set to 0 [13]. This algorithm is iterated for T number of iterations and the best set of features is returned.

## 4. The proposed method

**Fig 1:**
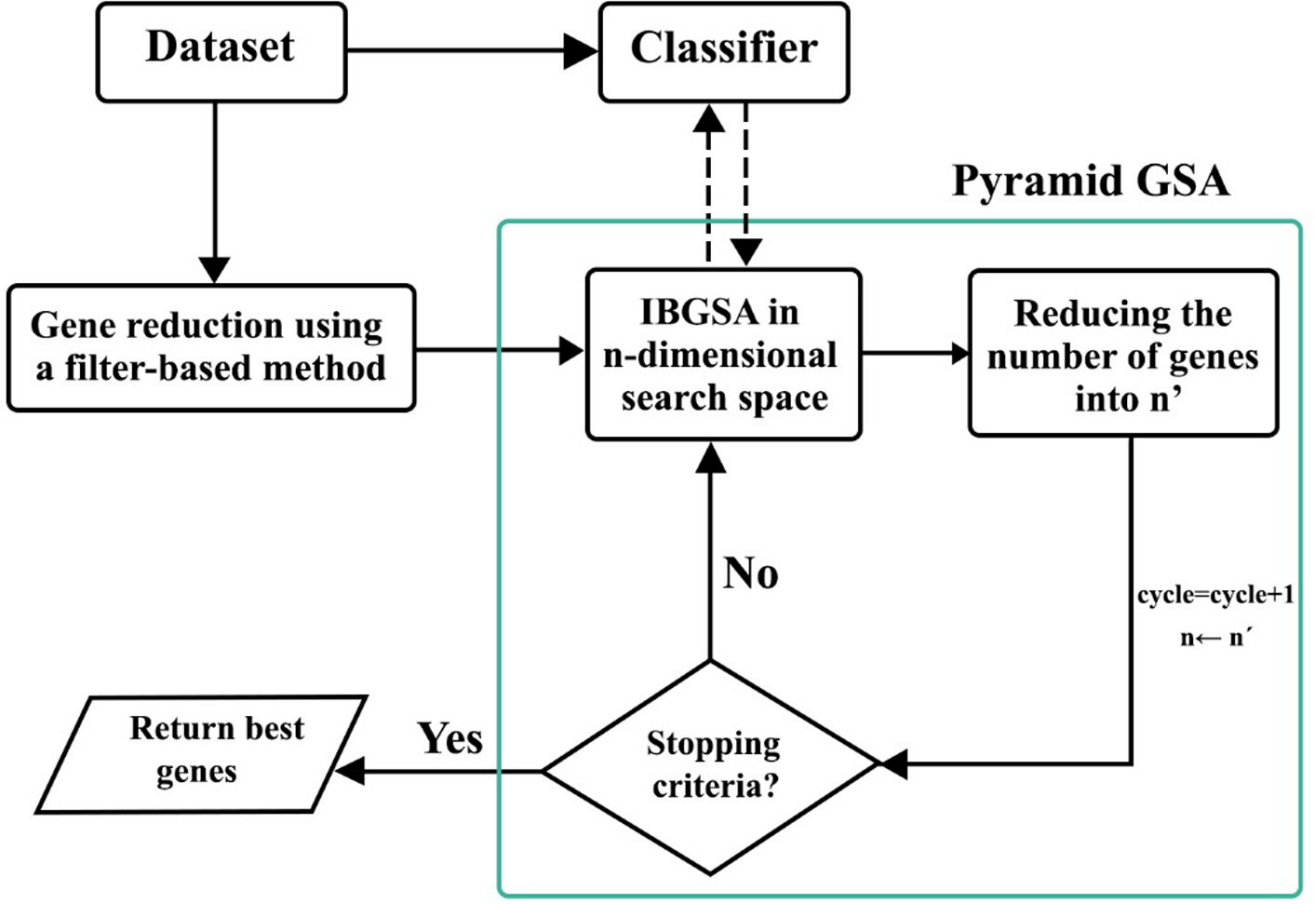
Gene selection using PGSA.

PGSA is a hybrid method which combined a filter and a wrapper method. The block diagram of PGSA is presented in Fig 1. The PGSA runs through several cycles to overcome the difficulties of high dimensionality. The method has two parts. At first, the number of genes is reduced by a filter-based method; then the gene set is passed on to the next step for further reduction. In the second step, the IBGSA is performed for some cycles. Final result of every cycle would have a lower number of features that is optimized according to the accuracy. The process will be repeated over several cycles in a way that the output of a certain cycle would be the input for the next cycle; thus, the number of genes and the dimension of search space will be reduced. In each cycle, PGSA works in joint with a classifier and try to maximize the classification accuracy. Each part of the algorithm would be dissected and explained thoroughly in the following sections.

### 4.1. Gene reduction using a filter-based method

In the first phase, genes are ranked using a filter-based method. We use the entropy characteristic for gene ranking. The high-ranked genes are selected and delivered to the following phase.

### 4.2. Gene reduction pyramidically using IBGSA

**Fig 2:**
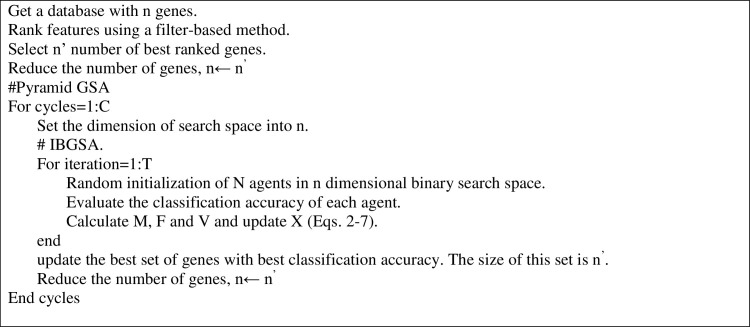
Pseudo code of gene selection using pyramid IBGSA.

The first phase of primary cycle reduces the number of genes into *n*. Then in the second phase, the number of genes is further reduced by IBGSA. The best genes are selected at each cycle, and the number of genes is updated for the next cycle. At the start of each cycle, the population of IBGSA is initialized randomly. The operators are then performed for some iterations and search the *n*-dimensional search space to find the best set of genes. After some iterations, the number of genes is reduced into n^’^ (n← n^’^). The next cycle is performed with the updated search space with n-dimension. With this method, the number of genes and the dimensionality of the search space are gradually reduced. At each cycle, IBGSA selects a subset of features that produces the best classification accuracy. The pseudo code is produced in Fig 2.

### 4.3. Model evaluation

We used two different methods to measure the performance of the algorithms. Firstly, we have divided the dataset into training (70%) and test (30%) subsets for the gene selection. when the most relevant genes were selected by the algorithms, we utilized MATLAB classification toolbox to model the selected genes obtained from the algorithms, and five-fold cross-validation has been used for evaluation. For the sake of simplicity, we have only shown the five-fold cross-validated results.

### 4.4. Experimental data

The dataset is based on microarray data of 20,545 gene expressions in 233 patients with breast cancer [47]. Six distinctive subtypes of breast cancer are provided that meticulously correlate with treatment response; each group’s characteristics are described separately in the Table 1. The dataset and additional information can be accessed through the GEO database (GSE20685). The data has been merged, normalized, batch effect-corrected for the preprocessing step, and filtered for genes with low variance via an integrated R pipeline [48].

**Table 1:** Characteristics and survival information for subgroups.

Subtype	patients	Characteristics	Approximate 12-month survival (%)
I	N = 37	Variable size	80
		Estrogen receptor (ER)-negative	
		Variable progesterone receptor (PR)	
		Her-2 negative	
		Low risk of distant metastasis	
II	N = 34	Large tumor	50
		ER-negative	
		Variable PR	
		Her-2 overexpression	
		High risk of distant metastasis	
III	N = 41	Large tumor	60
		Weak ER	
		Variable PR and Her-2	
		Low risk of distant metastasis	
IV	N = 40	Large tumor	50
		ER-positive	
		PR-positive	
		Her-2 overexpression	
		High risk of distant metastasis	
V	N = 41	Small tumor	85
		ER-positive	
		PR-positive	
		Her-2-negative	
		Least likely to distant metastasis	
VI	N = 40	Small tumor	80
		ER-positive	
		PR-positive	
		Her-2 negative	
		High risk of distant metastasis	

### 4.5. Benchmark algorithms

The results are compared with three heuristic search algorithms for gene selection using the FEATURESELECT software in MATLAB [49]. These methods are the following: Genetic algorithm (GA), particle swarm optimization (PSO) and imperialistic competitive algorithm (ICA). The SVM degree of kernel, gamma and tolerance of termination criterion were 3, 1 and 0.001 respectively.

The fitness function used by PGSA is defined as the classification accuracy as Eq 8. Accuracy, true positive rate (TPR), positive predictive value (PPV) and F_1_-score, are calculated as Eqs 9–11, respectively. Accuracy shows that how well the method correctly classified the samples. TPR indicates how well the method correctly classified positive samples. PPV is the probability that subjects with a positive test for a breast cancer subgroup genuinely have the correct one. F_1_-score is the harmonic mean of PPV and TPR. All statistical analyses were performed in MATLAB.


$$Accuracy=\frac{Number\;of\;Correct\;classified\;samples}{Total\;number\;of\;samples}$$ (8)

$$True\;Positive\;Rate\;\left(TPR\right)=\frac{TP}{TP+FN}$$ (9)

$$Positive\;Predictive\;Value\;\left(PPV\right)=\frac{TP}{TP+FP}$$ (10)

$$F_{1}score=2\times \frac{PPV\times TPR}{PPV+TPR}$$ (11)

## 5. Results

### 5.1. Workflow of feature selection

The best result of thirty independent runs are considered. For GA, PSO and ICA, the number of agents is set to 60. For the PGSA method, the total number of cycles is 8, the k1 is equal to one and k2 is 500. The total number of fitness evaluations is set to 480 for all algorithms.

The number of genes, the SVM kernel best suited for the model and each algorithm’s best accuracy will be provided in Table 2. All the computational processes were run on MATLAB 2021 with a Core i5 CPU and eight gigabytes RAM.

**Table 2:** The overall accuracy of gene selection algorithms.

*Algorithm*	Accuracy of the best model	Best SVM kernel	number of genes
**GA**	0.721	Quadratic	76
**PSO**	0.687	Quadratic	77
**ICA**	0.794	Cubic	76
**PGSA**	0.845	Quadratic	73

### 5.2. Feature selection benchmark

**Fig 3:**
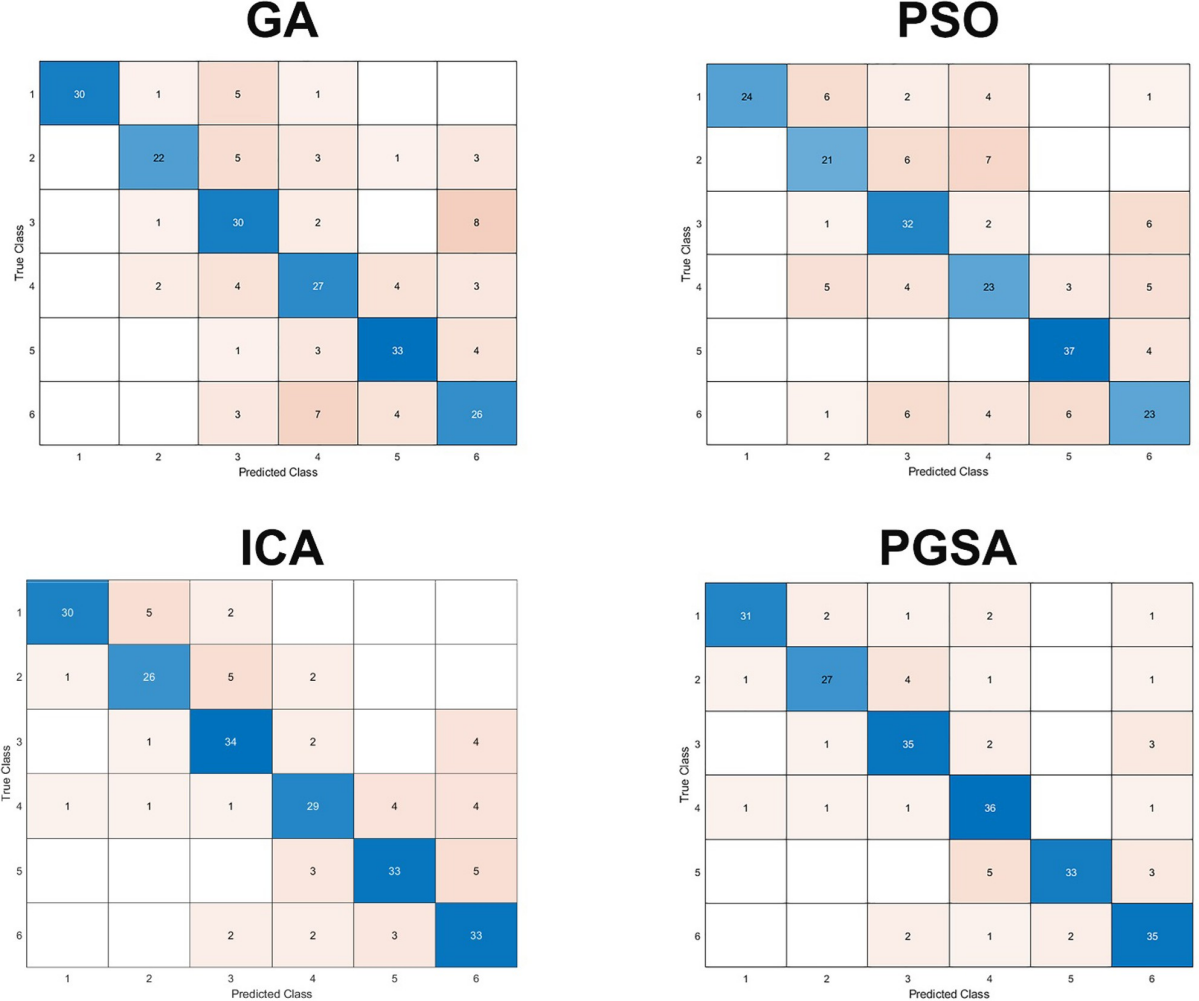
Confusion matrix of optimization algorithms.

**Fig 4:**
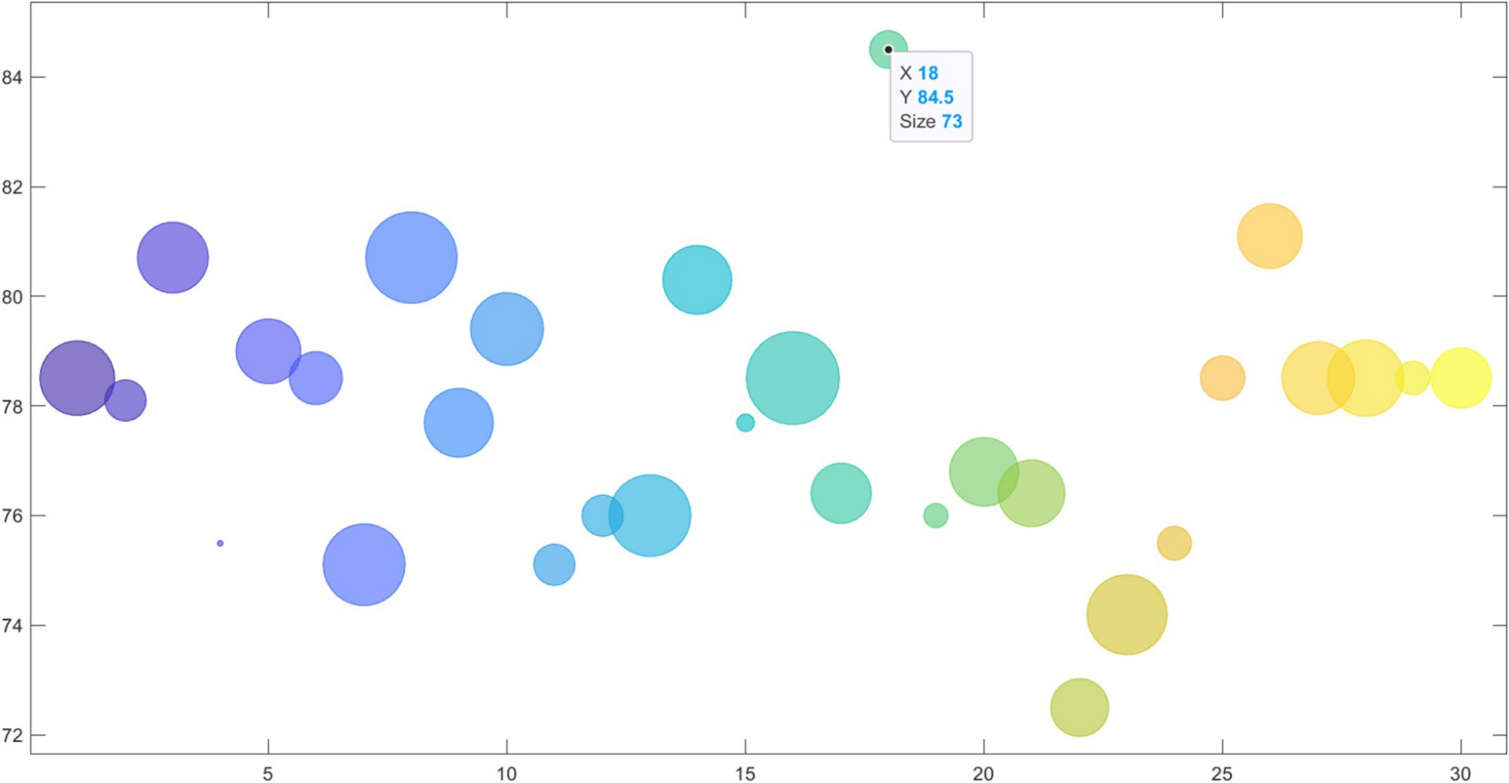
Accuracy (%) of PGSA during thirty independent runs. The Y and X axes imply the accuracy and iteration respectively. The bubble size is correlated with the number of genes; the bigger the bubble, the higher the number of genes. The best model was reached at the 18^th^ run with 84.5% accuracy and 73 genes.

The overall accuracy, TPR, PPV and F_1_-score of the best model during the five-fold cross-validation on the whole dataset are provided in Tables 2 and 3, respectively. As we can see, PGSA could reach the highest overall accuracy (84.5%) followed by ICA and GA. The PSO was the least accurate one (68.7%). Moreover, PGSA reduce the number of genes to 73 genes (i.e., approximately 280 times more compact than the original dataset dimension) which is lower than the other algorithms. It shows that PGSA could reduce the number of genes and maintain reasonably good accuracy. The confusion matrix of four optimization algorithms is shown in Fig 3. In case of TPR metric, there is a much more harmony in every class for PGSA (minimum TPR of 0.77 and maximum of 0.94 with standard deviation of 0.07 for PGSA) than others and it indicates the beneficence of PGSA in clinical context. In case of PPV, there is a higher variance in results for GA, PSO and ICA than PGSA (minimum of 0.79, maximum of 0.9 with standard deviation of 0.04 for PGSA) and it implies that by using PGSA, more patients will gain from the new classification. The Fig 4 shows the accuracy and number of genes during thirty runs.

**Table 3:** Five-fold cross-validated TPR, PPV, and F_1_-score of different algorithms. The best result for each class has been bolded.

	Class 1	Class 2	Class 3	Class 4	Class 5	Class 6
**TPR**						
**GA**	**1.0**	0.85	0.63	0.63	0.79	0.59
**PSO**	**1.0**	0.62	0.64	0.57	0.80	0.59
**ICA**	0.94	0.79	0.77	0.76	0.82	0.72
**PGSA**	0.94	**0.87**	**0.81**	**0.77**	**0.94**	**0.80**
**PPV**						
**GA**	0.81	0.65	0.73	0.68	0.80	0.65
**PSO**	0.65	0.62	0.78	0.57	**0.90**	0.57
**ICA**	0.81	0.76	0.83	0.72	0.80	0.82
**PGSA**	**0.84**	**0.79**	**0.85**	**0.90**	0.80	**0.88**
**F**_**1**_ **-score**						
**GA**	**0.90**	0.73	0.67	0.65	0.80	0.62
**PSO**	0.79	0.62	0.70	0.57	0.85	0.58
**ICA**	0.87	0.78	0.80	0.74	0.81	0.77
**PGSA**	0.89	**0.83**	**0.83**	**0.83**	**0.87**	**0.83**

### 5.3. Network analysis

**Fig 5:**
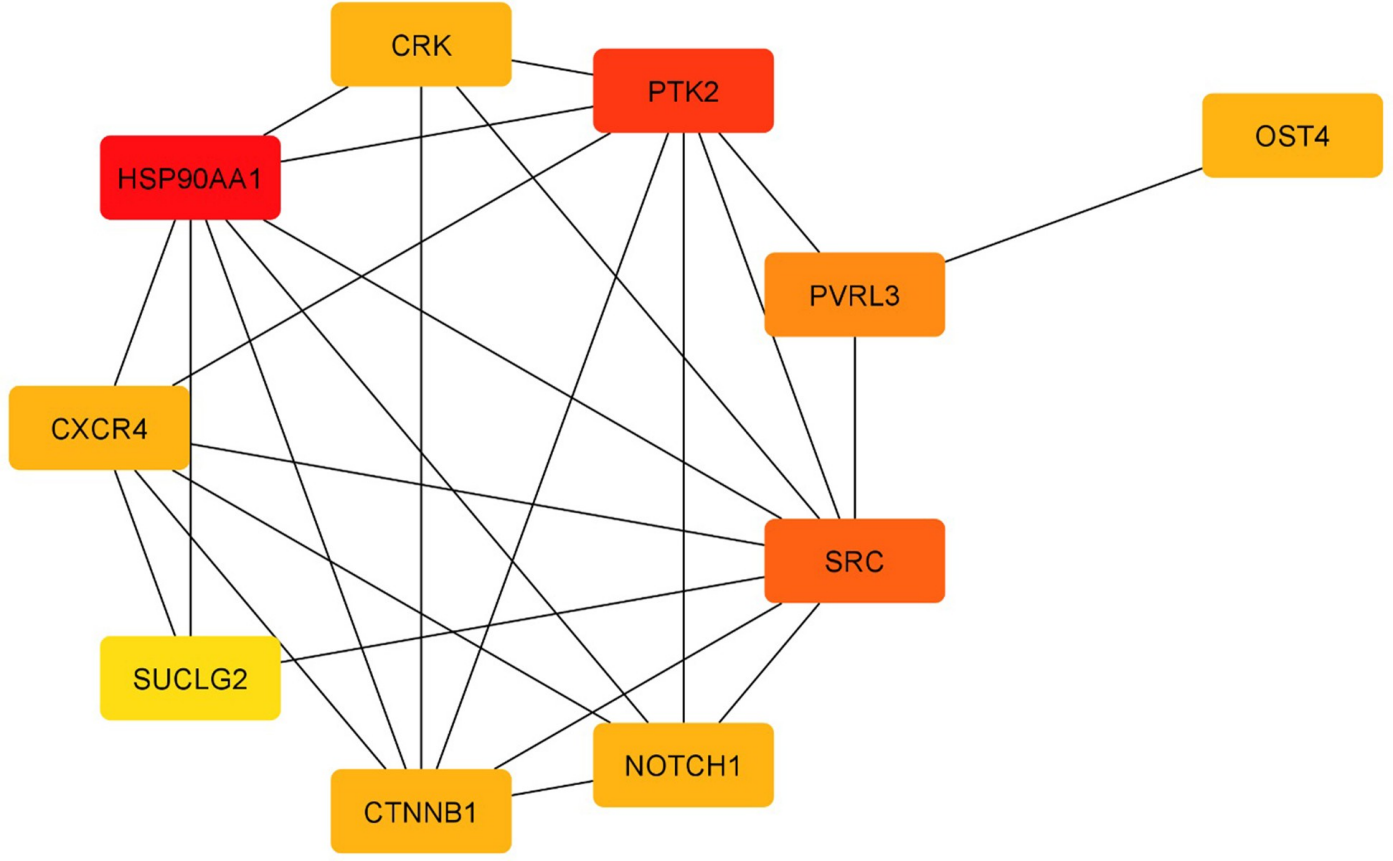
Bottleneck subnetwork constructed based on PGSA selected genes in breast cancer. Red and yellow colors indicate higher and lower bottleneck scores in the network, respectively.

To understand the interaction of selected genes, we constructed the protein-protein interaction (PPI) network using the STRING database. Maximum ten additional interactions with a confidence cut-off of 0.4 have been selected to retrieve the most crucial gene-gene (i.e., protein-protein) interactions. We used cytohubba extension in Cytoscape to find the top 10 genes with the highest bottleneck value in the network [50]. Bottlenecks are nodes in networks which is thought to be an indicator of essentiality for cell viability. The results are depicted in Fig 5. In the course of this work, we discovered that heat shock protein 90-alpha (HSP90AA1) is the most highlighted gene in the network and based on the available data, HSP90AA1 is an evolutionary conserved protein which has a prominent role in processes such as DNA damage, inflammation and tumorigenesis. there is a considerable body of evidence that shows plasma levels of HSP90AA1 has clinical benefit in prediction of onset and risk of metastasis in breast cancer patients [51]. In the present work, it also became apparent that HSP90AA1 may has a role in prediction of response to therapy in breast cancer. The next important bottleneck gene, is protein tyrosine kinase 2 (PTK2) which is an enzyme playing crucial roles in cell adhesion, migration and survival and aberrant upregulation of PTK2 in epithelial cells leads to malignancies such as breast cancer. Upregulation of PTK2 is correlated with poor survival and drug resistance in patients with breast cancer [52]. In concordance with previously mentioned genes, the SRC gene is involved in similar processes such as cell adhesion, migration, and survival. Moreover, there is a relationship between the SRC and estrogen receptor, which makes the SRC a novel source of investigation in response to therapy in tumors like breast cancer [53]. Results show that the PGSA method performs with sufficient reliability when used in genetic data for breast cancer.

## 6. Conclusion

PGSA, a hybrid feature selection method, was used with the goal of identifying the most important genes driving response to therapy in breast cancer. In comparison to GA, PSO and ICA, PGSA could reach to a lower number of genes while achieving an accuracy of 84.5 percent. From network analysis, we were able to deduce that the most critical genes involved in the prediction of response to therapy were those connected to DNA repair, inflammation, and cellular adhesion processes. The main characteristic of PGSA is the consistency of the selected genes, and these genes are in line with the prior discoveries in predicting breast cancer prognosis. To the best of our knowledge, there was no metaheuristic feature selection benchmark study on this dataset.

Thanks to recent breakthroughs in genomics and epigenetics, the etiology of diseases can be studied in great detail. Statistical methods for detecting the causes of the disease only independently analyze the different genetic and proteomic elements; the volume of data produced by genome-wide association study (GWAS) methods complicates the computational processes and takes a long time to achieve the ground truth solutions. personalized medicine must evolve quickly and reliable feature selection (i.e., gene selection) techniques that can shrink vast quantities of data is highly needed to develop; As a result, personalized genetic tests (PGTs) for each condition could be developed and made available to the public, greatly aiding in the screening, monitoring and predicting the response to therapy.

## Supporting information

S1 Graphical abstract (TIF)
