# Supplementary figures and images for: Gene selection using pyramid gravitational search algorithm
Source: PLoS One. 2022 Mar 15;17(3):e0265351. doi: 10.1371/journal.pone.0265351 (PMC8923457; doi:10.1371/journal.pone.0265351)

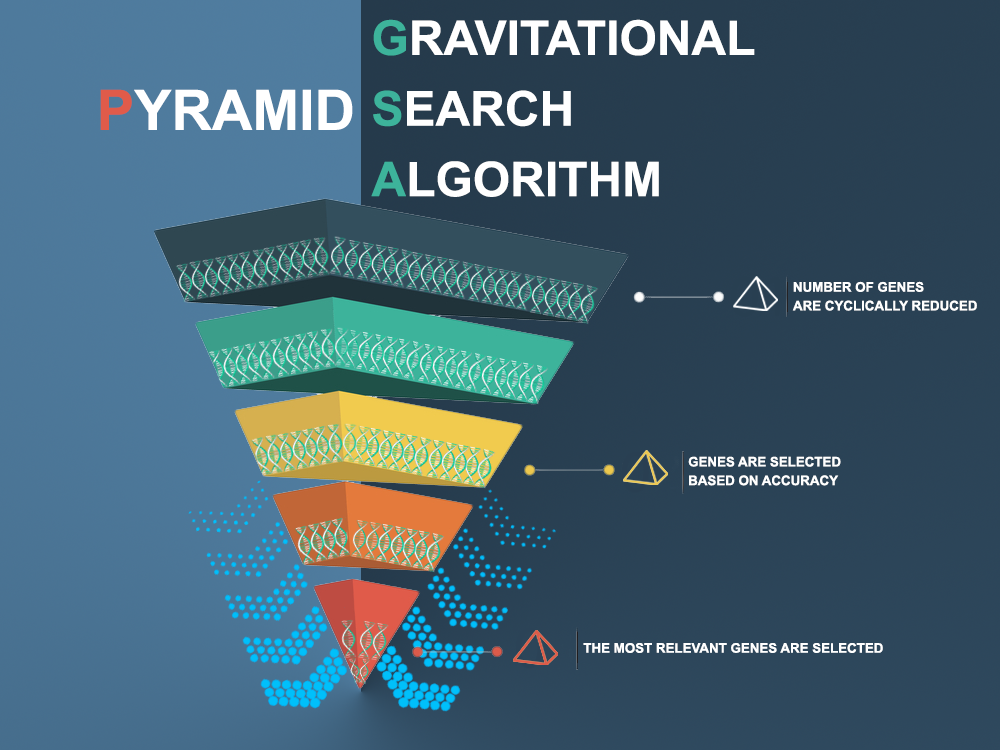

Supplement: S1 Graphical abstract — (TIF) [file pone.0265351.s001.tif]
